# Testing a longitudinal compensation model in premanifest Huntington’s disease

**DOI:** 10.1093/brain/awy122

**Published:** 2018-05-17

**Authors:** Sarah Gregory, Jeffrey D Long, Stefan Klöppel, Adeel Razi, Elisa Scheller, Lora Minkova, Eileanoir B Johnson, Alexandra Durr, Raymund A C Roos, Blair R Leavitt, James A Mills, Julie C Stout, Rachael I Scahill, Sarah J Tabrizi, Geraint Rees, Track-On investigators, A Coleman, J Decolongon, M Fan, T Koren, B Leavitt, A Durr, C Jauffret, D Justo, S Lehericy, K Nigaud, R Valabrègue, R Roos, E P ‘t Hart, A Schoonderbeek, C Berna, H Crawford, R Ghosh, D Hensman, E Johnson, P McColgan, M Papoutsi, J Read, G Owen, D Craufurd, R Reilmann, N Weber, I Labuschagne, B Landwehrmeyer, M Orth

**Affiliations:** 1 Huntington’s disease Research Centre, UCL Institute of Neurology, University College London, London, UK; 2 Wellcome Trust Centre for Neuroimaging, Institute of Neurology, University College London, London, UK; 3 Department of Psychiatry, Carver College of Medicine, University of Iowa, Iowa, City, IA, USA; 4 Department of Biostatistics, College of Public Health, University of Iowa, Iowa City, IA, USA; 5 University Hospital for Old Age Psychiatry, Murtenstrasse 21, 3010 Bern, Switzerland; 6 Department of Psychiatry and Psychotherapy, Medical Center, University of Freiburg, Freiburg, Germany; 7 Department of Electronic Engineering, N.E.D University of Engineering and Technology, Karachi, Pakistan; 8 Freiburg Brain Imaging Division, Medical Center, University of Freiburg, Freiburg, Germany; 9 Department of Neurodegenerative Disease, UCL Institute of Neurology, University College London, London, UK; 10 ICM - Institut du Cerveau et de la Moelle Epinière, INSERM U1127, CNRS UMR7225, Sorbonne Universités – UPMC Université Paris VI UMR_S1127and APHP, Genetic department, Pitié-Salpêtrière University Hospital, Paris, France; 11 Leiden University Medical Center, Department of Neurology, Leiden, The Netherlands; 12 Centre for Molecular Medicine and Therapeutics, Department of Medical Genetics, University of British Columbia, Canada; 13 School of Psychological Sciences and Institute of Clinical and Cognitive Neuroscience, Monash University, Melbourne, Australia; 14 Institute of Cognitive Neuroscience, University College London, London, UK

**Keywords:** compensation, neurodegeneration, Huntington’s disease, functional MRI

## Abstract

The initial stages of neurodegeneration are commonly marked by normal levels of cognitive and motor performance despite the presence of structural brain pathology. Compensation is widely assumed to account for this preserved behaviour, but despite the apparent simplicity of such a concept, it has proven incredibly difficult to demonstrate such a phenomenon and distinguish it from disease-related pathology. Recently, we developed a model of compensation whereby brain activation, behaviour and pathology, components key to understanding compensation, have specific longitudinal trajectories over three phases of progression. Here, we empirically validate our explicit mathematical model by testing for the presence of compensation over time in neurodegeneration. Huntington’s disease is an ideal model for examining longitudinal compensation in neurodegeneration as it is both monogenic and fully penetrant, so disease progression and potential compensation can be monitored many years prior to diagnosis. We defined our conditions for compensation as non-linear longitudinal trajectories of brain activity and performance in the presence of linear neuronal degeneration and applied our model of compensation to a large longitudinal cohort of premanifest and early-stage Huntington’s disease patients from the multisite Track-On HD study. Focusing on cognitive and motor networks, we integrated progressive volume loss, task and resting state functional MRI and cognitive and motor behaviour across three sequential phases of neurodegenerative disease progression, adjusted for genetic disease load. Multivariate linear mixed models were fitted and trajectories for each variable tested. Our conceptualization of compensation was partially realized across certain motor and cognitive networks at differing levels. We found several significant network trends that were more complex than that hypothesized in our model. These trends suggest changes to our theoretical model where the network effects are delayed relative to performance effects. There was evidence of compensation primarily in the prefrontal component of the cognitive network, with increased effective connectivity between the left and right dorsolateral prefrontal cortex. Having developed an operational model for the explicit testing of longitudinal compensation in neurodegeneration, it appears that general patterns of our framework are consistent with the empirical data. With the proposed modifications, our operational model of compensation can be used to test for both cross-sectional and longitudinal compensation in neurodegenerative disease with similar patterns to Huntington’s disease.

## Teaser

Owing to compensatory processes, the initial stages of neurodegeneration are marked by normal performance despite the presence of pathology. Using their explicit mathematical model, Gregory *et al.* report the first empirical examination of compensation over time in neurodegeneration, showing evidence of motor and cognitive compensation in a Huntington’s disease cohort.

## Introduction

Compensation is presumed to account for preserved behaviour in neurodegeneration during the initial stages of disease. Several studies have investigated this phenomenon (Kloppel *et al.*, 2009; Malejko *et al.*, 2014; Dillen *et al.*, 2016; Jones *et al.*, 2016; Poston *et al.*, 2016; Pan *et al.*, 2017), but none used a consistent definition of compensation. Consequently, a range of possible compensatory mechanisms has been proposed, often *post hoc*, including increased task-network brain activity or unexpected activity in an area not typically associated with performance of a particular behaviour. Thus, it is unclear to what extent reported changes in brain activity represent compensation or simply reflect the deleterious effect of disease-related pathology.

The lack of both a well-defined characterization of compensation and a corresponding model for empirical investigation has resulted in only a partial understanding of the nature of compensation in neurodegeneration. In response, we developed a model that operationalizes compensation providing a clearer and concise mathematical description of compensation (Gregory *et al.*, 2017). This model was based on one theory of compensation in healthy ageing whereby compensation is indexed by increased activation within an existing network (Barulli and Stern, 2013). As compensation occurs over time, we described a theoretical framework in which brain activation, behavioural performance, and structural volume loss have specific longitudinal trajectories over three phases of progression. Assuming that structural disease load increases steadily throughout, our model proposes that during the earliest phase, constant performance is maintained by increased brain activation; in the second phase, brain activation decreases as performance levels begin to deteriorate; and in the final stage, both brain activation and performance levels decrease rapidly, similar to brain volume. This verbal description is operationalized through statistical models, and evidence of compensation for a particular dataset is evaluated with parameter estimates and statistical tests.

Considering Huntington’s disease as our reference, specific longitudinal trajectory patterns are required for compensation. A compensatory relationship is indexed by longitudinal trajectories of brain activity and performance that are non-linear, specifically concave-down (with respect to the time axis), while structural disease load increases linearly over time (this scenario assumes that lower values of performance indicate greater deterioration). Our model requires an extended epoch in order to increase the likelihood that the non-linear trajectories will be observed (as opposed to only observing the increase in activation in the early stage, for example). In many observational longitudinal studies, there is a wide range of ages at study entry, which allows between-participant information to enhance within-participant change. Our model allows leveraging the combination of between- and within-participant information to evaluate longitudinal patterns of compensation.

In this study, we present the first empirical test of our compensation model in a neurodegeneration cohort. We consider both task functional MRI and resting state functional MRI markers of potential compensation in a Huntington’s disease cohort of premanifest (pre-HD) and early Huntington’s disease patients. Huntington’s disease is an ideal model for examining the nature of longitudinal compensation in neurodegeneration because it is both monogenic and fully penetrant caused by an expansion of the CAG triplet repeat in the *HTT* gene (full penetrance for CAG ≥ 40). There is a reliable genetic test for the Huntington’s disease gene mutation expansion and so it is possible to monitor disease progression and the onset of compensation many years prior to motor diagnosis. To anticipate our findings, we found evidence of motor compensation in Huntington’s disease indexed by connectivity between regions of the secondary motor cortex, but our results motivate extensions to our model to fully characterize patterns of compensation.

## Materials and methods

### Participants

A total of *n* = 110 participants were recruited from the four Track-On HD study sites (Kloppel *et al.*, 2015). All participants had different initial levels of progression but were all known Huntington’s disease gene mutation expansion carriers (94 pre-HD and 16 early Huntington’s disease). All participants were followed for 36 months with up to three annual visits (task functional MRI data were only collected for two time points at all sites). Most pre-HD participants came from the earlier Track-HD study (Tabrizi *et al.*, 2013). Those recruited specifically for the Track-On study were required to have a CAG repeat length ≥ 40 and a disease burden score (DBS) (Penney *et al.*, 1997) >250 at recruitment. Exclusion criteria included manifest disease, age below 18 or above 65 (unless previously in the Track-HD study), major psychiatric, neurological or medical disorder or a history of severe head injury (for full details see Kloppel *et al.*, 2015). For the statistical analysis, there was up to 303 observations; 89 participants (81%) had three visits (repeated measurements), 15 (14%) had two visits, and six (5%) had one visit. Missing data varied among variables, and the number of participants and observations are noted for each analysis. Only right-handed people were included in the analysis, and one clear outlier was excluded who had almost the smallest brain volume but was relatively young and had almost the shortest CAG expansion. The study was approved by the local ethics committees and all participants gave written informed consent according to the Declaration of Helsinki.

### Behavioural measures and functional MRI tasks

For the resting state functional MRI analyses, a global cognitive composite score was derived from nine cognitive tasks that were completed in testing sessions separate to the MRI procedures [Stroop Word Reading test, Symbol Digit Modality Test, Paced Tapping, Circle Tracing (two conditions), Map Search test, Cancellation task, the Spot the Change task, Mental Rotation task] (for full details see Kloppel *et al.*, 2015)*.* Based on the Track-HD study the Unified Huntington’s Disease Rating Scale (UHDRS)-Total Motor Score (TMS), and Quantitative Motor (Q-Motor) Speeded Tapping were selected as markers of motor performance (for full information see Kloppel *et al.*, 2015).

Participants also performed a Verbal Working Memory (VWM) and Sequential Finger Movement (SFM) functional MRI task in the scanner. For the VWM task, participants performed a verbal *n*-back task with two levels of working memory load (1-back and 2-back) whereby they were required to respond according to whether the letter on screen was the same as the letter presented one letter previously (1-back) or presented two letters previously (2-back). Performance in the 1-back and 2-back conditions was analysed using the *d*-prime coefficient (probability of correct response minus probability of false positive responses). For the SFM task, participants performed a motor task that involved metronome-paced finger tapping with their right (dominant) hand (see Kloppel *et al.*, 2015 for a detailed description). Mean timing inaccuracies (cue-response intervals) and standard deviations for four conditions comprising all permutations of complexity (simple/complex) and speed (slow/fast) were included in the compensation model as independent outcome variables for all task functional MRI analyses. All motor variables were natural log transformed to make their empirical distribution more symmetric. Procedures and variable selections were identical to the cross-sectional study on compensation (Kloppel *et al.*, 2015).

### MRI data acquisition

3T MRI data were acquired on two different scanner systems (Philips Achieva at Leiden and Vancouver and Siemens TIM Trio at London and Paris) as described for each of the three visits (Tabrizi *et al.*, 2013; Kloppel *et al.*, 2015). For task and resting state functional MRI, whole-brain volumes were acquired at a repetition time of 3 s using a T_2_*-weighted echo planar imaging (EPI) sequence with the following parameters: echo time 30 ms, field of view 212 mm, flip angle 80°, 48 slices in ascending order (slice thickness: 2.8 mm, gap: 1.5 mm, in plane resolution 3.3 × 3 mm) and bandwidth of 1906 Hz/Px. For resting state functional MRI, 165 volumes were acquired over 8:20 min followed by field map acquisition. Two hundred and twenty-five volumes over 11:15 min for the SFM task and 190 volumes over 9:30 min of VWM task functional MRI data. For the third visit due to the time constraints of the scanning sessions, the tasks were each performed at only two sites: the SFM task was performed at Vancouver and Leiden only; the VWM back task was performed at Paris and London only. The missing data were accounted for in our statistical analyses (see below). Standardization of data acquisition across sites was performed based on previous suggestions (Kloppel *et al.*, 2015).

### MRI data processing

T_1_-weighted images were processed as described in Kloppel *et al.* (2015). The brain disease load composite score was derived from the segmentations of the structural MRI data and included whole brain grey matter, white matter, caudate and putamen volumes (all corrected for total intracranial volume). The volumes from all three time-points for each of the four segmentations were included in a principal components analysis with the resulting weights providing the basis for the brain disease burden score. For further details see the online Supplementary material.

Functional MRI data preprocessing and subsequent statistical analyses were performed using SPM8 running under MATLAB for each of the three visits (Kloppel *et al.*, 2015). The T_1_ scan was segmented into grey and white matter using the VBM8 toolbox (http://dbm.neuro.uni-jena.de/vbm/) and used to create an improved anatomical scan for coregistration. Using the DARTEL extension, deformation parameters were extracted for normalization of functional images (Ashburner and Friston, 2011). The first four EPI images were discarded to allow for steady state equilibrium. Functional images were first realigned and field maps used for inhomogeneity correction whenever available. For resting state functional MRI, all EPI images were then coregistered to the new anatomical image and normalized using DARTEL deformation parameters. For task functional MRI, only contrast images were normalized and smoothed. Finally, data were smoothed using a 6 mm full-width at half-maximum Gaussian kernel. See our previous study for a description of standardization and quality control measures (Kloppel *et al.*, 2015).

### MRI data analysis

We performed task-based functional MRI analyses based on preselected regions of interest identical to those used in the resting state functional MRI analyses (see below). A first-level analysis based on the general linear model (GLM) was performed for each participant on the smoothed images. Task-related blood oxygen level-dependent signal changes were estimated for each task condition. Six head movement regressors were also modelled, in addition to the instruction screen, single button presses during rest and blocks during which participants performed a wrong condition for the motor task. For the regions of interest, we used peaks identified in the analyses of the task-specific main effect. Only data from the first two visits were used to avoid biasing the peak by the sites that contributed three visits. The 2-back versus 1-back contrast (VWM) and the complexity (complex > simple) and speed (fast > slow) contrasts (SFM) were included in the compensation model. Brain signals were extracted from task-network regions of interest and included in the model as possible compensator variables; associated performance was included as the corresponding behavioural variable.

Resting state functional MRI data were analysed using two complementary connectivity analysis techniques: seed-region based correlation (functional connectivity) and dynamic causal modelling (DCM; effective connectivity) (Friston *et al.*, 2003). For the functional connectivity analyses, seed-based analysis was used to identify temporal correlations (or functional connectivity) between activity within *a priori* selected regions in the left and right dorsolateral prefrontal cortex (DLPFC) (cognitive) (Owen *et al.*, 2005) and the left primary motor network (motor) and activity within every voxel across the whole brain (for further details, see Kloppel *et al.*, 2015). Signal values for significant correlations were extracted and included in our model as compensators. For the effective connectivity analyses, DCM uses a model-based approach to examine brain connectivity, measuring the directed effects of activity in one region on another region. Regions for the network models were derived from the baseline task-functional MRI analyses (Kloppel *et al.*, 2015). As such, both structural brain volume and behavioural measures support our original model in that they follow a concave-down trajectory of changes over time, but several of the putative compensator variables displayed a significant cubic rather than quadratic longitudinal trajectory. We suggest, however, that even in these cases, these examples partly fulfil our criteria for compensation due to the concave-down pattern occurring in the latter stage. Ultimately, we recommend expanding our theoretical model of compensation to capture greater complexity in the brain activity trends.

All biologically plausible directed connections between five regions within the cognitive network and seven regions within the motor network were modelled (Supplementary material).

### Compensation model

**Figure 1:**
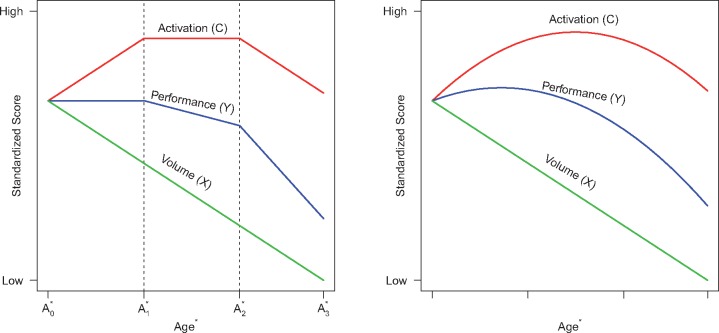
**Compensation model and polynomial approximation.** *Left*: The compensation model. *Right*: The polynomial approximation. Dashed vertical lines denote phases of compensation. Age* is age-adjusted for CAG expansion.

As illustrated in Fig. 1, the main components of compensation are a performance outcome (Y), an activation signal compensator (C), and brain volume (X), which are tracked over time. The time metric for the longitudinal analysis represents a Huntington’s disease-appropriate transformation of age (denoted as $Age^{*}$). Huntington’s disease is caused by a CAG expansion, and the longer the expansion the earlier the motor onset (Ross and Tabrizi, 2011). To account for this acceleration, age can be adjusted for CAG expansion, which enables the tracking of individuals with various CAG expansions on the same time metric (i.e. age adjusted for CAG expansion; see below for details). The vertical axis represents scores on measures standardized to have equal means at the first observation. Three phases are depicted spanning ${\text{Age}}_{0}^{*}$ to ${\text{Age}}_{1}^{*}$ (Phase 1), ${\text{Age}}_{1}^{*}$ to ${\text{Age}}_{2}^{*}$ (Phase 2), and beyond ${\text{Age}}_{2}^{*}$ (Phase 3). For Huntington’s disease, brain volume is expected to steadily decrease over time regardless of phase, which is consistent with research findings (Paulsen *et al.*, 2014). Phase 1 is compensation, where brain activation increases in reaction to brain deterioration, and the increased activation causes performance to be maintained. In Phase 2, disease effects start to overwhelm compensation, which results in an activation plateau and the initiation of performance deterioration. Phase 3 shows relentless disease effects with brain activation starting to decrease and performance deterioration accelerating.

**Figure 2:**
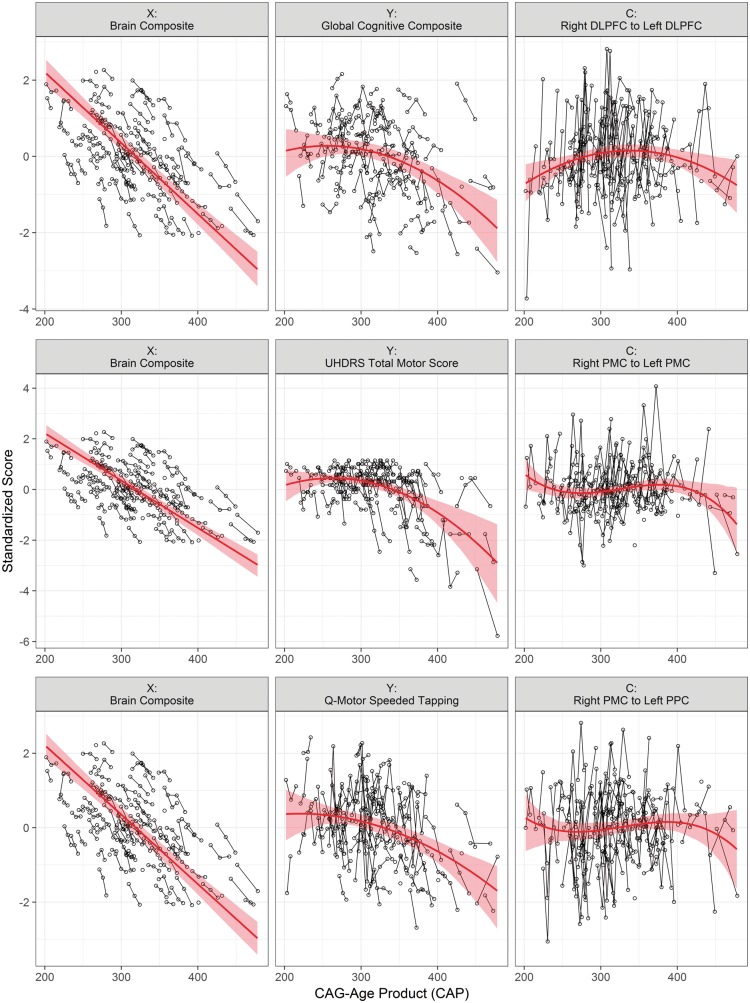
**An example of compensation in the secondary motor cortex.** Standardized empirical data (circles and thin lines) and fitted curves (thick lines) with 95% bootstrap confidence intervals, for three sets of variables. The first column shows the disease load variable, brain volume composite; the column shows the behavioural variable, and the third column shows the compensator variable Total motor score was multiplied by −1 prior to standardization, and smaller values indicated greater deterioration. PMC = premotor cortex; PPC = posterior parietal cortex.

The right panel of Fig. 2 shows approximations that result when quadratic polynomial regression models are fitted to the data of the left panel. The performance and compensator curves are approximated by a quadratic curve; whereas brain volume is approximated by a linear curve. Under a polynomial regression analysis, the right panel curve patterns suggest criteria for determining if empirical data are consistent with Huntington’s disease compensation. Using the polynomial curves as our touchstone, we propose that the following three conditions are necessary for consistency with compensation: (i) brain volume (X) shows a linear decrease over time (as patients age); (ii) the performance variable (Y) has a concave-down pattern over time; and (iii) the compensator variable (C) also has a concave-down pattern over time.

When regression models are used for the analysis, the above conditions translate into the following statistical results. Condition 1: a statistically significant negative linear trend for X. Condition 2: a significant negative quadratic trend for Y (the quadratic regression coefficient must have a negative sign for the curve to be concave-down). Condition 3: a significant negative quadratic trend for C. If the previous conditions hold, then we also expect a slower downward acceleration for *C* relative to Y at some late-phase point, but this is not formally tested. The difference in acceleration is difficult to test because its effect will be small relative to testing against the null of the three conditions (it is common for statistical power to be greater when testing if a regression coefficient is statistically different from 0 then testing the difference of coefficients) (Dupont and Plummer, 1998). Therefore, the difference in acceleration is not formally included as a condition. A rigorous evaluation of the conditions involves the possibility that each variable could have a simpler curve or more complex curve than proposed in the conditions. Therefore, evaluation of each condition will involve fitting linear, quadratic, and cubic trend models.

### Statistical analysis

The compensation analysis involved three correlated variables (X, Y, C) measured on the same individuals over time. To account for the correlation between variables and within time, multivariate linear mixed models (MLMMs) (Verbeke, 2000) were used for the analysis (multivariate refers to multiple outcome variables). The analysis involved one brain volume composite variable (X), eight candidate performance variables (Y), and 61 candidate compensator variables (C). The performance motor variables were multiplied by −1, so that smaller values on all performance variables represented greater deterioration, which was consistent with the X and C variables.

Because of the well-known relationship between the timing of motor onset and CAG expansion (Lee *et al.*, 2012), it was important to account for CAG expansion. An approach that has proved useful in Huntington’s disease research (Paulsen *et al.*, 2014) is to adjust age for CAG expansion by means of the CAG-Age Product (CAP) transformation (Ross *et al.*, 2014). The time metric of the analysis was CAP=age×(CAG−35.5), which is similar to the disease burden score of Penney *et al.* (1997), and adjusts age for the acceleration due to CAG expansion. To facilitate estimation and testing with the MLMMs, CAP was centred using M=320 (the approximate mean among participants and repeated measures) and scaled by S=60 [the approximate standard deviation (SD)], which we denote as $CAP^{*}=(CAP-320)/60$. Variables were on different scales, and to facilitate comparison, each variable was standardized based on its own vector of scores (among participants and repeated measures). The standardization meant that change over time for each variable was expressed in standard deviation units (for all variables, smaller scores indicated greater deterioration).

A rigorous evaluation of the compensation conditions requires that a trajectory simpler than the hypothesized trajectory be ruled out, and a trajectory more complex also be ruled out. For example, the second condition hypothesizes a quadratic trajectory for *Y*, and a rigorous demonstration would rule out the simpler linear trajectory and the more complex cubic trajectory. For this reason, we fit linear, quadratic, and cubic polynomial models for the first three compensation conditions.

Let $Z_{ij}^{\left(v\right)}$ denote the standardized score for the $v^{th}$ variable (v=X,Y,C) for the $i^{th}$ participant ($i=1,\ldots ,N_{v}$) and the $j^{th}$ time point ($j=1,\ldots ,n_{i}$). Then the single-variable equation of the most complex cubic MLMM is,

(1) $$Z_{ij}^{\left(v\right)}=\alpha^{\left(v\right)}+a_{i}^{\left(v\right)}+\beta_{1}^{\left(v\right)}(CAP^{*})+\beta_{2}^{\left(v\right)}{\left(CAP^{*}\right)}^{2}+\beta_{3}^{\left(v\right)}{\left(CAP^{*}\right)}^{3}+\gamma^{\left(v\right)}{\text{x}}_{i}^{T}+e_{ij}^{\left(v\right)}$$

where the Greek letters indicate fixed effects for the $v^{th}$ variable, $\alpha^{\left(v\right)}$ is the fixed intercept, $a_{i}^{\left(v\right)}$ is the random intercept, and $e_{ij}^{\left(v\right)}$ is random error. For an individual outcome, the two random variables are assumed to be uncorrelated, and each is assumed to be normally distributed with zero-mean and non-zero variance. ${\text{x}}_{i}$ is a vector of dummy variables for sex, education (ISCED level 3 or 4 or 5), and site (London, Leiden, Paris, Vancouver), with associated fixed effects vector γ. The quadratic model omits the cubic term and the linear model additionally omits the quadratic term. When the MLMM involves two or three outcome variables—bivariate with $Z_{ij}^{\left(X\right)}$ and $Z_{ij}^{\left(Y\right)}$, and trivariate with $Z_{ij}^{\left(X\right)}$, $Z_{ij}^{\left(Y\right)}$, $Z_{ij}^{\left(C\right)}$—the random effects are allowed to correlate. (Details of specifying the bivariate and trivariate MLMMs are provided in Thiebaut *et al.*, 2002; Long, 2012; Gregory *et al.*, 2017.)

The MLMM was estimated using restricted maximum likelihood (REML). When there are missing data (as in this analysis), REML yields unbiased estimates under the assumption that the missing data mechanism is ignorable. Testing was performed using the Wald statistic (z-ratio), which was the ratio of a regression coefficient to its standard error (SE). To account for multiple testing, the false discovery rate (FDR) q-value was computed, along with the Wald P-value. Adjustment was made based on the grouping of variables explained below. Statistical significance was defined by the criterion q<0.05 (except for the first set of tests).

Because of a varying number of candidate variables, a three-step strategy of statistical testing was used, beginning with a univariate LMM for X only, then bivariate MLMMs with X and Y, and finally trivariate MLMMs with X, Y, and C. In the first step, the univariate model was estimated for brain volume in isolation (not considering Y and C) because it was the only candidate X variable. Consistent with the first compensation condition, the null hypothesis of $H_{0}:\beta_{3}^{\left(X\right)}=0$ (zero cubic coefficient) was tested with the cubic polynomial model, $H_{0}:\beta_{2}^{\left(X\right)}=0$ (zero quadratic coefficient) was tested with the quadratic model, and $H_{0}:\beta_{1}^{\left(X\right)}=0$ was tested with the linear model. Significance was determined by P-values for these tests. As the results below show, testing revealed that a linear model for the brain volume composite was sufficient, and the quadratic and cubic models for X were no longer considered.

In the second step, each Y variable was modelled along with the X variable in a bivariate MLMM, with a linear curve specified for X. The series of tests for linear, quadratic, and cubic terms was conducted for *Y*. FDR adjustment was based on the group of eight Y variables. The statistically significant Y variables (q<0.05) were then used in the trivariate MLMMs (non-significant variables were not considered further). As the results below show, testing revealed that a quadratic model was sufficient for every *Y*, and only the quadratic model was carried forward.

The third step involved estimation of a trivariate MLMM with X (linear curve), Y (quadratic curve), and C. The series of tests for linear, quadratic, and cubic terms was conducted for *C*. The previous two steps resulted in filtering out several *Y* variables, so that the trivariate models consisted of a subset of 59 *Y* and *C* variable combinations. The FDR adjustment was based on the group of C variables with a common Y variable. For each *Y* variable in the analysis, there were several associated compensator variables. For example, the *Y* variable of global cognitive composite had 28 associated connectivity variables. The FDR correction was performed for the group of compensators for each *Y* variable. So, in this case, the FDR correction was applied to the 28 tests in the trivariate analysis. Regarding the informal (i.e. not tested) difference in acceleration between the *Y* and *C* variables, we had to allow for the possibility of a significant cubic effect or a significant quadratic effect (see ‘Results’ section). Therefore, the instantaneous rate of change at the late point of CAP=420 was computed, with this value being the 0.95 percentile of the CAP scores. The instantaneous rate of change is the first derivative of the significant polynomial model at CAP = 420.

## Results

### Compensation conditions

#### Compensation Condition 1: linear increase of structural disease load (brain volume)

Brain volume composite (*X*) was examined testing the linear fixed effect coefficient, the quadratic coefficient, and the cubic coefficient in separate models. The linear model for brain volume had a coefficient estimate (SE) of ${\hat{\beta }}_{1}^{\left(X\right)}$ = −1.0271 (0.0509), and the Wald test was statistically significant (*P* < 0.0001). The quadratic model for brain volume was not statistically significant, ${\hat{\beta }}_{2}^{\left(X\right)}$ = −0.0289 (0.0276), *P* = 0.2963, and the cubic model also was not significant, ${\hat{\beta }}_{3}^{\left(X\right)}=0.0263\backslash \left(0.0158\right),P=0.0954$. The results provide strong evidence that brain volume change can be characterized by a decreasing linear trend over CAP. Therefore, the linear curve for brain volume was used in all subsequent analyses.

#### Compensation Condition 2: non-linear decrease of cognitive and motor behavioural variables

Behavioural variables for the cognitive and motor networks, for both task (*Y*) and resting state (*C*) analyses were combined in our second analysis (Table 1). Table 1 displays the results for all behavioural variables from the bivariate MLMM (modelled with linear change in brain volume). UHDRS-TMS, Q-Motor Speeded Tapping, the global cognitive composite and two conditions of the SFM task (slow simple and slow complex) had significant negative quadratic coefficients (concave-down trend) *q*’s < 0.05, whereas the remaining variables had significant negative linear (decreasing) trends. The behavioural variables showing consistency with our model conditions, UHDRS-TMS, Q-Motor Speeded Tapping, the global cognitive composite and the most significant condition from the SFM task (slow simple), were included in the next step of trivariate modelling.

**Table 1:** Performance variable (*Y*) results based on the bivariate linear mixed model

*Y* variable	Linear	Quadratic	Cubic	Slope	*n*	Obs
UHDRS Total Motor Score	−0.6530 (0.0739)***	−0.2168 (0.0491)***	−0.0035 (0.0354)	−1.2721 (0.1583)	**107**	**288**
Q-Motor Speeded Tapping	−0.4852 (0.0759)***	−0.1195 (0.0536)*	0.0553 (0.0391)	−0.8300 (0.1720)	**107**	**288**
Global Cognitive Composite	−0.4268 (0.0696)***	−0.1711 (0.0458)***	−0.0449 (0.0292)	−0.9095 (0.1471)	**107**	**288**
Speeded Tapping (slow, simple)	−0.1902 (0.0892)*	−0.1729 (0.0702)*	−0.0992 (0.0529)	−0.7432 (0.2402)	**96**	**193**
Speeded Tapping (fast, simple)	−0.1692 (0.0668)**	−0.0369 (0.0535)	−0.0316 (0.0409)	−0.2870 (0.1835)	96	193
Speeded Tapping (slow, complex)	−0.2718 (0.0826)***	−0.1375 (0.0637)*	−0.0558 (0.0488)	−0.7133 (0.2194)	**96**	**193**
Speeded Tapping (fast, complex)	−0.1616 (0.0774)*	−0.0225 (0.0619)	−0.0608 (0.0471)	−0.2335 (0.2120)	96	193
Verbal Working Memory (d-prime)	−0.2078 (0.0892)*	−0.0039 (0.0682)	−0.0223 (0.0532)	−0.2199 (0.2285)	97	217

*q*-values are not provided for the slope because it is not used for inference (only description); *q < 0.05, **q < 0.01, ***q < 0.001.

Results for the performance variables from the bivariate MLMM (modelled with linear change in brain volume composite). The table lists the estimated polynomial trend coefficient estimate (SE), and significant level. and associated inferential statistics, along with the sample size and total number of observations (Obs). Significant results are highlighted in bold.

#### Compensation Condition 3: concave-downward trend for compensator variables

Based on the analyses for compensation Condition 2, the following pairings of behavioural and compensatory variables were used in our trivariate analysis (brain volume composite was always an additional variable in the model). For the motor network: UHDRS-TMS and Q-Motor Speeded Tapping were separately used as behavioural variables with DCM connectivity parameters as compensators; SFM performance was used as the behavioural variable with SFM task-based brain activity as compensator. For the cognitive network: global cognitive composite was used as the behavioural variable for seed-based functional connectivity and DCM connectivity parameters. Table 2 shows the statistically significant results for the test of polynomial coefficients in the separate trivariate models (full results are shown in Supplementary Table 1).

**Table 2:** Significant trivariate model results

*Y* variable	*C* variable	Linear	Quadratic	Cubic	Slope
UHDRS Total Motor Score	Effective connection from left PMC to right PMC	0.0179 (0.0639)	−0.0612 (0.0502)	−0.0850 (0.0384)*	−0.4365 (0.2033)
UHDRS Total Motor Score	Effective connection from right PMC to left PMC	−0.0160 (0.0632)	−0.0884 (0.0497)	−0.1244 (0.0375)**	−0.6785 (0.1987)
UHDRS Total Motor Score	Effective connection from right PMC to left PPC	0.0205 (0.0725)	−0.0303 (0.0561)	−0.0951 (0.0422)*	−0.3619 (0.2233)
Q-Motor Speeded Tapping	Effective connection from left PMC to right PMC	0.0143 (0.0639)	−0.0591 (0.0504)	−0.0846 (0.0387)*	−0.4358 (0.2053)
Q-Motor Speeded Tapping	Effective connection from right PMC to left PMC	−0.0205 (0.0634)	−0.0858 (0.0499)	−0.1229 (0.0379)**	−0.6750 (0.2008)
Q-Motor Speeded Tapping	Effective connection from right PMC to left PPC	0.0202 (0.0726)	−0.0293 (0.0564)	−0.0921 (0.0428)*	−0.3536 (0.2262)
Global Cognitive Composite	Effective connection from left DLPFC to right DLPFC	0.0167 (0.0682)	−0.1504 (0.0523)*	0.0639 (0.0397)	−0.4376 (0.1709)
Global Cognitive Composite	Effective connection from right DLPFC to left DLPFC	0.0719 (0.0684)	−0.1769 (0.0515)**	0.0787 (0.0389)	−0.4643 (0.1685)
Global Cognitive Composite	Functional connection between left DLPFC and right lateral occipital cortex	0.0522 (0.0725)	−0.0486 (0.0561)	0.1315 (0.0413)*	0.3060 (0.2174)

*q*-values are not provided for the slope because it is not used for inference (only description); *q < 0.05, **q < 0.01, ***q < 0.001. PMC = premotor cortex; PPC = posterior parietal cortex.

#### Compensation in the cognitive and motor networks

Table 2 shows there were two variable combinations consistent with the conditions of compensation, i.e. where both behaviour and compensator variables showed a quadratic trend: global cognitive composite combined with the left DLPFC to right DLPFC connection and the right DLPFC to left DLPFC connection. For the remaining combinations, the compensator variables, i.e. the brain connectivity measures showed significant cubic trends. The last column of Table 2 shows that all the cubic trends did have a decreasing instantaneous slope for the late CAP value (CAP = 420), except for the connection between the left DLPFC and right lateral occipital cortex. A visualization of the results is shown in Fig. 2; the circles connected by thin lines indicate the individual data points for each sampled time point, and the red curves indicate the fitted polynomials from the models with 95% bootstrapped confidence intervals. The first row depicts one compensator variable with a quadratic trend (right DLPFC to left DLPFC connection), and the second and third rows show two compensator variables with cubic trends. The other cubic trends were similar, except for the connection between the left DLPFC and right lateral occipital cortex (Table 2).

## Discussion

In the current study we have empirically investigated longitudinal compensation in premanifest Huntington’s disease using an explicit model of compensation. Using a novel framework previously proposed (Gregory *et al.*, 2017) we examined longitudinal compensation in cognitive and motor networks in Huntington’s disease with up to 3 years of data. Our model integrated progressive brain volume loss, task and resting state functional MRI derived markers of compensation, and cognitive and motor behaviour to test for the presence of compensation across three sequential phases of neurodegenerative disease progression. We hypothesized that compensation would be evidenced by linear decline in brain volume, but a non-linear concave-down pattern of both brain activity and behaviour, with brain activity eventually declining at a slower rate and maintaining normal behaviour. Consistent with our model, we found that these compensation conditions were fulfilled by combinations of variables involving the global cognition and the cognitive network, and partially fulfilled by combinations of variables that involved the motor network and both clinical and quantitative motor performance. More specifically, global cognition was temporarily maintained by increased effective connectivity between the left and right dorsolateral prefrontal cortex, such that the connections (as compensators) showed significant concave-down patterns, while both clinical UHDRS Total Motor Score and quantitative motor behaviour were supported by increased connectivity between the left and right premotor cortex. However, in the latter case, the concave-down patterns occurred within significant cubic trends, i.e. later than proposed in our model and so only partially fulfilling our prespecified criteria for compensation. As such, our original model requires some revision to allow for connectivity effects to be delayed relative to behaviour effects.

Compensation primarily confers advantage to neurodegenerative patients by facilitating maintenance of normal behaviour in the presence of pathology. Accordingly, our conceptualization of compensation was realized across both the motor and cognitive networks at differing levels. We primarily identified evidence of compensation in the cognitive network using effective or directed connectivity parameters between the left and right DLPFC, with the global cognitive composite as the behavioural variable. Thus, we provide evidence that connectivity was increased between the left and right hemispheres in the presence of ongoing decline in brain volume. Cognitive deficits are prominent in Huntington’s disease gene carriers many years prior to onset (Paulsen *et al.*, 2008; Tabrizi *et al.*, 2012, 2013). However, given that some gene carriers present with more motor signs, others more cognitive signs, the exact nature of the mechanisms underlying these deficits is unknown. The DLPFC is a region that is commonly involved in higher-order cognitive processing, including tasks that are components of our global cognitive composite, such as the Stroop Word Reading and the Symbol Digit Modality tests. We previously tested compensation in Huntington’s disease using a simplified cross-sectional approach, focusing solely on participants with the highest structural disease load. We showed that individuals with higher disease load displayed increased activity within the right parietal cortex and increased connectivity between the right DLPFC and regions within the left hemisphere to maintain cognitive performance (Kloppel *et al.*, 2015). It is important to stress that in the current study we explicitly model the longitudinal trajectory of compensation, with no *a priori* knowledge regarding patterns of longitudinal compensation for either cognitive or motor networks. However, it is nonetheless compelling that we have identified a pattern of longitudinal compensation in the cognitive network involving the DLPFC. Collectively, these findings suggest that the DLPFC plays a role in maintaining cognitive function in pre-HD that is consistent over time. It is particularly interesting that the strongest effect was seen in the connection from the right to left DLPFC, compatible with the right-sided compensation effects that we identified previously and indicating that the DLPFC may be an important target area for investigation in terms of improving cognitive deficits in Huntington’s disease gene carriers.

A similar pattern of compensation was also evident in the motor network for connections between the left and right premotor cortex and the right premotor and parietal cortices when using both the clinically-based UHDRS Total Motor Score and the Q-Motor behavioural measure of Speeded Tapping. However, these patterns did not completely fulfil our hypothesized criteria for compensation as the compensating variables, i.e. connectivity parameters, showed a concave-down pattern as part of a cubic rather than quadratic trend. We have postulated previously that for compensation to be present, both performance and compensatory brain activity should follow a concave-down trajectory over time, such that both will peak and gradually deteriorate over time but with the trajectory for brain activity slower to progress than that of behaviour to maintain normal levels of behaviour (Gregory *et al.*, 2017).

We accept that brain activity could simply represent Huntington’s disease-related pathology independent of the compensatory process, but believe that we have shown that our model reflects functional MRI activity as an index of compensation. However, it is possible that brain activity may follow a more complex trajectory compared to that of behaviour and structural disease load. Patterns of change in brain activity may vary across different brain regions, due to variability in the timing of the cellular effects of the huntingtin gene mutation. In the case of the motor network, for example, motor deficits in Huntington’s disease become evident around the point of disease onset and as such are used in the process of clinical diagnosis. There is considerable evidence to suggest volumetric differences in primary and premotor regions of the motor cortex in Huntington’s disease when compared to controls (Kassubek *et al.*, 2004; Wolf *et al.*, 2009; Dogan *et al.*, 2013). This is supported by new work using structural DCM that investigates longitudinal change in grey matter cortical volume over a 7-year period in premanifest and recently diagnosed early-Huntington’s disease gene carriers (in preparation). Here, motor brain regions including bilateral primary, premotor and supplementary motor undergo a fairly rapid period of accelerated atrophy in the years shortly after clinical diagnosis; this is in contrast to other brain regions, e.g. in the frontal lobe where atrophy is more pronounced in the premanifest stages. It is therefore possible that motor brain activity is more variable during the premanifest stages of disease, leading to what appears to be a delayed compensatory effect and made evident as a cubic trend in Fig. 2. The trajectory of brain activity does appear to become more consistent as disease progresses, however, with all three examples in Fig. 2 indicating that around the point where the CAP score reaches 400, there is deterioration in brain activity for both networks that occurs at a slower pace than behavioural change.

Overall, the results provide evidence that compensation does not follow one canonical course and as a central concept may incorporate a number of compensatory processes that act concurrently. Furthermore, it is also possible that our approach will not necessarily identify all conceivable patterns of compensation such as those that are only apparent under certain cognitive conditions and that other models of reserve and compensation as discussed in the healthy ageing literature may be more appropriate. In the current study, we have adapted our original model (Gregory *et al.*, 2017), to consider the possibility that each type of variable may have a simpler or more complex curve than the theoretical conditions we initially proposed necessary to justify compensation. As such, both structural brain volume and behavioural measures support our original model in that they follow a concave-down trajectory of changes over time, but several of the putative compensator variables displayed a significant cubic rather than quadratic longitudinal trajectory. We suggest, however, that even in these cases, these examples partly fulfil our criteria for compensation due to the concave-down pattern occurring in the latter stage. Ultimately, we recommend expanding our theoretical model of compensation to capture greater complexity in the brain activity trends.

Conceptualizing longitudinal compensation presents a major challenge for the neurodegeneration field. It can be characterized by a number of differing models of cognitive reserve or compensation, which focus on an increase in activation, as modelled here, a decrease in activation or simply a slowing of deterioration. Furthermore, compensatory and disease effects may not vary sufficiently to be detectable over a relatively short period. Our approach here has been to develop and test a model encompassing both within- and between-participant changes. As our data spanned 3 years, longitudinal compensation patterns were largely inferred from between-participant differences. Differences in individual rates of disease progression may obscure some of the longitudinal changes in compensation. It will be optimal to collect a long time series (over many years or indeed decades) so that compensation processes can be observed within-participant, but resources for such a study would be considerable. Nonetheless, we have developed an operational model of compensation that can now be used in this way to test for both cross-sectional and longitudinal compensation in other neurodegenerative disease with similar patterns to Huntington’s disease.

## Supplementary Material

Supplementary Data

## Abbreviations

CAP: CAG-Age Product
DLPFC: dorsolateral prefrontal cortex
MLMM: multivariate linear mixed model
pre-HD: premanifest Huntington’s disease
SFM: Sequential Finger Movement
UHDRS: Unified Huntington’s Disease Rating Scale
