# Supplementary material for: Testing a longitudinal compensation model in premanifest Huntington’s disease
Source: Brain. 2018 May 17;141(7):2156–66. doi: 10.1093/brain/awy122 (PMC6022638; doi:10.1093/brain/awy122)

# Supplementary Information

## Supplementary Methods

### *Brain Disease Load Composite Score Calculation*

| all visits | Factor Pattern (Correlations) | | | | |
|---|---|---|---|---|---|
| | | Factor1 | Factor2 | Factor3 | |
| | gm2icv | 0.64116 | -0.5643 | 0.51755 | |
| | wm2icv | 0.6213 | 0.66446 | 0.4153 | |
| | caud2icv | 0.85028 | 0.00877 | -0.3924 | |
| | putamen2icv | 0.87554 | -0.0668 | -0.2926 | |
| | | | | | |
| Standardized Scoring Coefficients | | | | | |
| | Factor1 | Factor2 | Factor3 | | Ratio to Grey Matter |
| gm2icv | **0.28039** | -0.7382 | 0.76117 | | **1** |
| wm2icv | **0.27171** | 0.86916 | 0.6108 | | **0.969043** |
| caud2icv | **0.37185** | 0.01147 | -0.5771 | | **1.326189** |
| putamen2icv | **0.38289** | -0.0874 | -0.4304 | | **1.365562** |

*Principal Components Analysis of the four structural measure included in the brain disease load composite score*

The brain disease burden score is a slightly simplified version of Factor 1:

$$\frac{4(\text{caudate2icv} - 0.419364)}{0.0692471} + \frac{3(\text{gm2ICV} - 0.450948)}{0.0342494} + \frac{4(\text{putamen2icv} - 0.499226)}{0.0903647} + \frac{3(\text{wm2icv} - 0.311182)}{0.0200908}$$

### *Region of Interest selection for Effective Connectivity analyses*

"For the DCM analyses all selected regions were empirically-derived and have been discussed previously (Kloppel *et al.*, 2015). For the cognitive network, the regions included were left DLPFC (-42, 32, 30), right DLPFC (42, 32, 30), left inferior parietal cortex (-34, -48, 38), right inferior parietal cortex (38, -46, 38) and the anterior cingulate cortex (ACC) (-2, 12, 42). These regions were largely selected to be compatible with our task-based analysis centred on a working memory task and the co-ordinates used to create our regional masks were selected according to a meta-analysis on working memory studies (Owen *et al.*, 2005). For the motor network, we selected regions of interest associated with motor behaviour and that have been shown to be involved in motor tapping in HD; the co-ordinates used to create our regional masks were selected according to a previous study in HD (Kloppel *et al.*, 2009). These included the left M1 (-40, -18, 60), the left premotor cortex (PMC) (-24, 0, 54 ) the right PMC (26, -6, 52), the pre supplementary motor area (SMA) ( 0, 6, 54), the caudal SMA (-6, -10, 54) the left superior parietal cortex (-22, -68, 58), and the right superior parietal cortex (22, -66, 60)."

**Supplementary Figures**

**Compensation networks based on our current findings**

*a) Motor Network showing increased bidirectional activity between the left and right premotor cortex and from the right premotor cortex to the left parietal cortex b) Cognitive Network showing increased bidirectional activity between the left and right DLPFC. ACC: anterior cingulate cortex; DLPFC: dorsolateral prefrontal cortex; PMC: premotor cortex; PPC: posterior parietal cortex; and SMA: supplementary motor area.*

***a)***

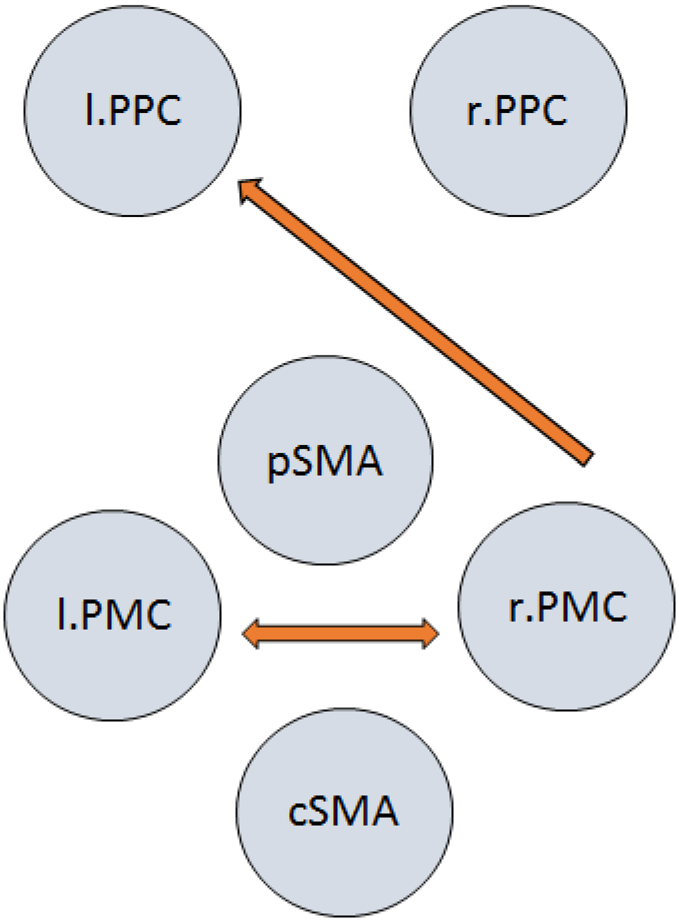

*b)*

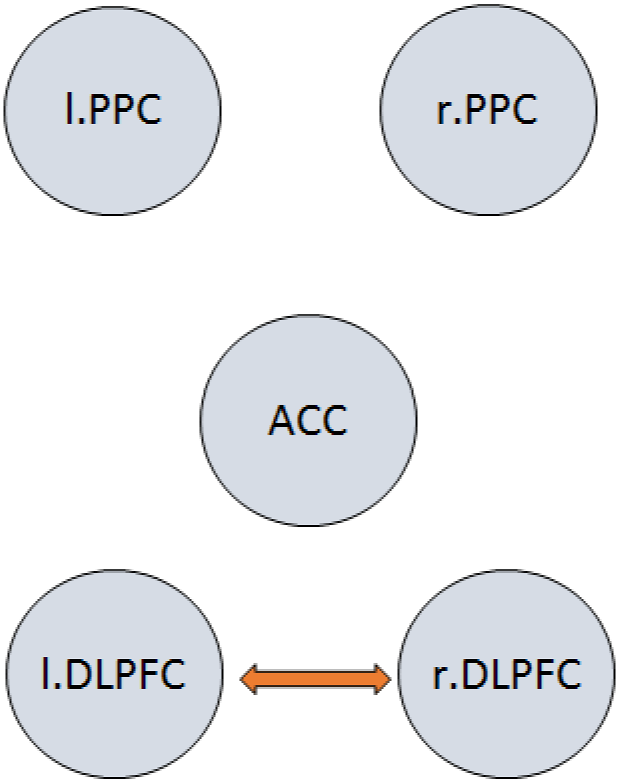

Supplement: Supplementary Data [file awy122_suppl_data.pdf]
